# Autonomy and infant feeding decision-making among teenage mothers in a rural and urban setting in KwaZulu-Natal, South Africa

**DOI:** 10.1186/s12884-018-1675-7

**Published:** 2018-02-17

**Authors:** Ngcwalisa Amanda Jama, Aurene Wilford, Lyn Haskins, Anna Coutsoudis, Lenore Spies, Christiane Horwood

**Affiliations:** 1 Centre for Rural Health, University of KwaZulu-Natal, Durban, South Africa; 2 Department of Pediatrics & Child Health, School of Clinical Medicine, University of KwaZulu-Natal, Durban, South Africa; 3 Department of Health, Pietermaritzburg, South Africa

**Keywords:** Teenage mother, Autonomy, Infant feeding practices, Decision making, Child nutrition, South Africa

## Abstract

**Background:**

The nutritional status of infants born to teenage mothers can be sub-optimal compared to those born to older mothers. One contributing factor is inappropriate feeding practices adopted by teenage mothers. Little is known about how infant feeding decisions are made among teenage mothers, particularly in under resourced settings. In this study we prospectively explored autonomy and infant feeding decision-making among teenage mothers in a rural and urban setting in KwaZulu-Natal, South Africa.

**Methods:**

This study adopted a qualitative longitudinal design. Thirty pregnant participants were recruited to the study cohort, from the catchment area of two hospitals (one urban and one rural). Participants were purposively selected to include teenagers, HIV positive, and working pregnant women. We report findings from ten teenage mothers, aged between 15 and 19 years, who participated in the larger cohort (*n* = 5 rural; *n* = 5 urban). Monthly in-depth interviews were conducted with participating mothers for 6 months starting 2 weeks after delivery. All interviews were conducted in the local language, transcribed verbatim and translated into English. Data was coded using NVivo v10 and framework analysis was used.

**Results:**

Findings from this study showed that teenage mothers had knowledge about recommended feeding practices. However, our findings suggest that these mothers were not involved in infant feeding decisions once they were at home, because infant feeding decision-making was a role largely assumed by older mothers in the family. Further, the age of the mother and financial dependency diminished her autonomy and ability to influence feeding practices or challenge incorrect advice given at home. Most feeding advice shared by family members was inappropriate, leading to poor infant feeding practices among teenage mothers. Returning to school and fear of breastfeeding in public were also barriers to exclusive breastfeeding.

**Conclusion:**

Teenage mothers had a limited role in the infant feeding decision-making process. Health workers have an important role to play in ensuring that knowledge about infant feeding is shared with the mother’s family where infant feeding choices are made. This will improve support for teenage mothers, and may also positively impact on the nutritional status of children.

## Background

There is high rate of teenage pregnancy in South Africa (SA) [1]. Teenage pregnancy is defined as a pregnancy in a girl aged under 20 years, and despite the availability of free contraceptive services, most of these pregnancies are unintended [2]. Sexual risk-taking behaviour, including early sexual debut, unprotected sex, multiple sex-partners and low contraceptive use are common among young people in SA [3]. The most recent SA demographic health survey, conducted in 2016, showed that 16% of girls aged between 15 and 19 years had begun childbearing, with KwaZulu-Natal (KZN) province among the highest, where 19% of teenage girls in this age group were either pregnant or had given birth [4].

Teenage pregnancy is often associated with poor outcomes for both the teenage mother and her baby [5–8]. Optimal infant feeding, particularly exclusive breastfeeding (EBF) for 6 months, is the most important determinant of infant health, development and wellbeing [9]. Therefore, it is crucial that teenage mothers adopt optimal feeding practices. Yet, young mothers may breastfeed less than older mothers [10]. Reasons for this include, lack of parenting readiness and dependence on social support systems that are not supportive of breastfeeding [3, 11]. In addition, pregnancy often occurs among teenagers when they are still at school and, in many cases, childbirth is followed by mothers returning to education environments, leaving the responsibility of childcare to their own parents, aunts, grandmothers and other family members [3]. In SA, teenage mothers are most likely to be living with their families, usually their own mother, and not with the father of the child [12].

The developmental phase and age of teenage mothers means that they often are dependent on their mother or other close family members for guidance and support [13]. In turn, teenagers actively seek guidance from their families, and friends [8]. Consequently, after the baby is born, older family members may assume the role of extending parenting from the teenager to the newborn. The mothers’ family are usually the primary source of financial and emotional support in the post-partum period when support from partners may decrease. Support from family friends and partners are among the most important factors affecting young mothers’ feeding choices [3].

Most infant feeding research includes mothers of all ages, and those studies that have focused specifically on teenage mothers and infant feeding decision-making have usually been conducted in developed countries [14–16]. We are unaware of any studies examining determinants of infant feeding practices among teenage mothers in a South African setting, or among African families. It is important to understand these issues to facilitate the design of fitting interventions to promote breastfeeding and improve feeding practices among teenage mothers in our setting. This study investigated autonomy and infant feeding decisions amongst teenage mothers in one rural and one urban setting in KZN.

## Methods

A longitudinal qualitative design was used to explore mothers’ narratives about infant feeding choices and practices from birth to 6 months of life, in order to prospectively capture critical processes involved in participants’ infant feeding choices during this period [17].

### Study site

This study was conducted in two sites in KZN, one urban and one rural site, selected in partnership with the KZN Department of Health. The rural site was located in Northern KZN and houses over 120,000 people, scattered over a 60 km radius, served by one district hospital. The urban site was in the greater Durban area with over 290,000 residents living in largely formal settlements, served by one district hospital in the area. The proportion of the facility-based deliveries among girls aged below 18 years, as routinely measured by the District Health Information System (DHIS), was 10.9% in the rural district and slightly lower at 7.5% in the urban district in 2016 [18].

### Study sampling

Women aged 15 years or older who were more than 36 weeks pregnant, were approached for recruitment in one antenatal clinic at each site. A purposive sampling technique was used to ensure inclusion of teenagers (aged≤19 years), working women and HIV infected women, in order to capture a diversity of experiences, beliefs and practices. This sampling strategy was employed to enhance our understanding of infant feeding decisions by including perspectives of participants from groups who were known to have different feeding practices [3, 19, 20].

**Fig. 1:**
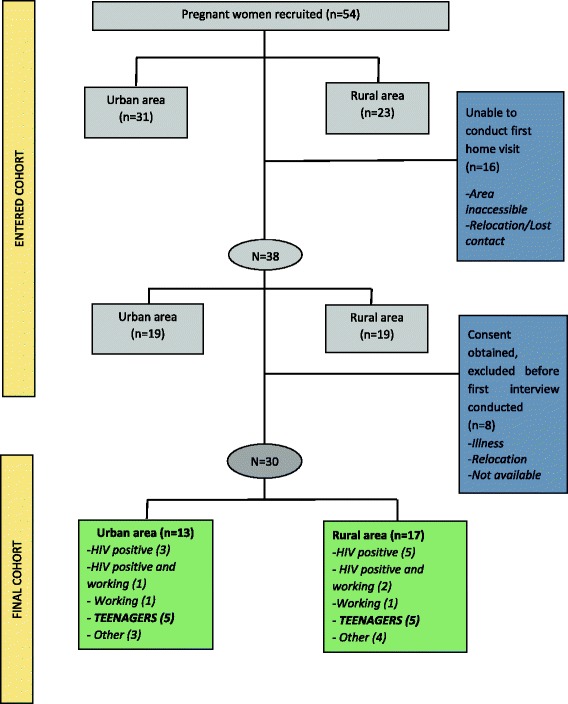
Participants sampling and cohort profile

Participants who did not plan to reside in the area with the baby for the first 6 months after delivery were excluded from the study. Eligible women were given a brief overview of the study and provided their contact details, they were then contacted by fieldworkers to arrange a home visit. At this visit, recruitment was completed and informed consent obtained for the whole study period. For teenagers aged below 18 years consent was obtained from the parent or guardian, and participants provided written assent. Ten pregnant teenagers were recruited, five per site, and these formed part of the larger cohort (Fig. 1).

### Data collection

This qualitative prospective study was undertaken between November 2015 and October 2016. Enrolled teenagers were visited in their homes during pregnancy to obtain consent. Baseline data was collected at this visit, including socioeconomic characteristics of the participants and their plans for feeding of the baby. Once participants had given birth, two trained field-workers, one per site, conducted monthly in-depth-interviews in the participants’ home for period of 6 months (total six interviews per participant). An in-depth interview guide was used to explore three main topics; current feeding practices; reasons for adopting particular feeding practices; people and situations that influenced infant feeding decisions; contacts with the health service. At each visit permission for privacy was sought from family members to allow for a safe environment where teens could freely express themselves.

Interviews were conducted in participants’ language of preference (either IsiZulu, IsiXhosa or English) and were audio recorded. For participants who had returned to school, interviews were conducted on school free days, such as weekends or holidays. The interview process involved an overview of the previous interviews, so the field workers would begin each interview with a recap of what was previously discussed. This technique allowed fieldworkers and participants to keep the focus on longitudinal elements of infant feeding practices and decision making [20].

### Ethics approval

Ethics approval for this study was obtained from the Biomedical Research Ethics Committee at the University of KwaZulu-Natal (BE301/15) and the KZN Department of Health. All participants signed a consent form before voluntarily participating in the study. For participants who were younger than 18 years, written consent was obtained from a parent or legal guardian and written assent from the participant. To preserve anonymity, codes were assigned to each participant based on the area, type of participant and number of the visit. Audio recordings and transcripts were stored at the University of KZN in a password protected file.

### Data analysis

All in-depth interviews were transcribed verbatim and translated into English. Framework analysis was used. This is a qualitative analysis method that aims to order data to reduce data volume and prioritize questions, and has been successfully and widely used by other authors [21–23]. We chose this analysis method to provide coherence, structure and a systematic approach to analysis of this large dataset. In addition, given the longitudinal nature of the data, this method allows between- and within-case comparisons [24], and provides a systematic way to reduce the data while still retaining the thick description and complexity [25]. This process is followed by interpretation of data using thematic analysis, typologies and explanation analysis. Analysis was based on pre-determined research themes (drawn from the interview guide) as well as inductive themes that emerged from the interview data. This approach highlighted the longitudinal as well as linear aspect of participants’ responses. Guided by the principles of framework analysis, the study team decided on particular research questions to prioritize the focus of the analysis. Transcripts were exported to NVivo v10 for coding, where themes were identified. Comparative analysis between two skilled qualitative researchers was performed to ensure analysis accuracy. Analysis was performed until no further themes emerged.

## Results

Ten teenagers between the ages of 15–19 were enrolled in the cohort. All teenagers were living with at least one adult in the household. Most of the teenagers were having their first child (Table 1). The analysis revealed four main themes: a) process of infant feeding decision-making in the household, b) poor infant feeding advice from key family members, c) autonomy and teenage dependency, d) managing conflict between clinic and home infant feeding advice. Direct quotes from participants are used to demonstrate relevant findings. Each teenage mothers was allocated an identifier, which can be seen in Table 1, for example (Teen 1, rural and the month of the interview which corresponds to the baby age at the time). Text inside square brackets [] represent the interviewer’s descriptions.

**Table 1:** Participants’ profile

Teen 1	Teen 2	Teen 3	Teen 4	Teen 5	Teen 6	Teen 7	Teen 8	Teen 9	Teen 10
Demographic characteristics of mothers									
Setting: Rural	Setting: Rural	Setting: Rural	Setting: Rural	Setting: Rural	Setting: Urban	Setting: Urban	Setting: Urban	Setting: Urban	Setting: Urban
Age: 16 years	Age: 18 years	Age: 17 years	Age: 19 years	Age: 16 years	Age: 18 years	Age: 16 years	Age: 16 years	Age: 15 years	Age: 18 years
Year of schooling: 10	Year of schooling: 11	Year of schooling: 8	Year of schooling: 9	Year of schooling: 9	Year of schooling: 11	Year of schooling: 9	Year of schooling: 9	Year of schooling: 10	Year of schooling: 12
Pregnancy #									
First	First	First	Second	First	Second	First	First	First	First
Antenatal feeding intentions for the first 6 months									
Mixed feeding	Exclusive breastfeeding	Breastfeeding and formula feeding	Exclusive breastfeeding	Exclusive breastfeeding	Exclusive breastfeeding	Exclusive breastfeeding	Exclusive breastfeeding	Exclusive breastfeeding	Exclusive formula feeding
No of people living in the household									
3 adults, 4 children	6 adults, 2 children	3 adults, 5 children	6 adults, 5 children	1 adult, 5 children	5 adults	1 adult, 1 child	5 adults, 3 children	6 adults, 3 children	2 adults, 2 children
Mothers source of financial support at follow up									
Grandmother; Child grant	Grandmother	Mother; Aunt	Child grant; Baby’s father	Mother	Grandmother; Baby’s father	Aunt	Mother; Baby’s father	Mother	Mother

### Infant feeding intentions and rationale

Among ten pregnant teenagers, eight expressed their intention to EBF their infants for 6 months. Participants stated that this intention was mainly based on their knowledge about benefits of EBF, from information which they received during Antenatal Care (ANC) visits and in the post-natal ward. Teen 7 reported:

*I chose to breastfeed her breast-milk only because after I had finished giving birth, the nurse told me not to give the baby anything except milk, excuse me, except breast milk or if I get medicine from the clinic that is all. She said I must not give the baby water and other things until she is 6 months old (Teen7, urban_month1).*

However, for some teens, not having money to buy formula was the reason given for why they wanted to breastfeed. Teen 5 mentioned: *I chose to breastfeed because for now I don’t have money to buy formula milk (Teen 5, rural_month1).*

Teen 10, who planned to formula feed only, stated that being HIV positive was the main reason for choosing to feed her baby formula: “*It’s too risky, the chance of him contracting it [HIV] uh it great. They [nurses] might not say it that great but I don’t want…I don’t want to take a chance”.* When visited postnatally it was observed that the mother did not initiate breastfeed and baby was fed formula and cereal at month one. This was the only teenage mother who was HIV positive in the study.

The one teen who planned to formula feed and breastfed never mentioned the reason for choosing this feeding method, but during follow-up visits it was observed that she was a school-going mother.

### Process of infant feeding decision making in the household

During the follow-up period, it emerged that mothers had introduced liquids or solids to the infants’ diet as early as within the first few days after the baby was born. Introduction of pre-lacteal feeds was found to be a common practice, with mothers saying that they introduced other fluids while they were waiting for their milk to come in, as advised by their own elders: “*Granny advised me to buy formula, because the milk wasn’t coming out enough and the baby was always crying. My granny felt she might catch cancer if she’s not feeding” (Teen1, rural_month1).*

Most infant decision making in the household were taken by elders with teenagers having little or no influence on how their babies are fed. For instance, when teenagers were asked who makes the decision on what the baby should eat in the household, 9/10 teenagers answered that it was either the grandmother, mother, aunt or older sister who made such decisions as stated by the teenager here:

*M: It is my mother who makes decisions on what the baby should eat and she keeps them. We only add our opinions there and there but really it is my mother (Teen 5, rural_month4).*

The decision making process was similar across households. They involved an elder assessing what the baby’s needs were, communicating these to the mother and telling the mother the solution. The mother was then expected to passively comply with this advice. Below, a teenage mother who introduced liquids and solids within the first month of birth describes:

*My mother advised me to add other foods because she ‘felt’ the baby wasn’t getting full from breastmilk only, so she advised me to give her soft foods like purity and yoghurt. Because with breast milk alone, she feeds frequently, but when she is given soft foods she the baby get full for a long time (Teen 1, rural_month1).*

Another teenager who changed from breastfeeding to formula feeding reported:

*P: They said I don’t have milk in my breasts. No matter how much I eat it doesn’t come out well*

*I Ok “they said” who was that?*

*P: My grandmother (Teen 2, rural_month1).*

At times, the elders changed what the baby ate based on their belief that the baby is old enough to experiment with other foods.

*P: They said I must stop him [from eating porridge] because he’s fine now*

*I: Who is ‘they’?*

*P: It was my aunt (Teen 6, urban_month3)*

Even when the teenagers noted the babies’ needs before the elders, most of them emphasized that they never made any feeding decisions alone without consulting an elder. Teen 4, who made decisions alone, was the exception to this. She was slightly older than the rest in the cohort, she planned to EBF and rejected any advice in the household that went against that. Her autonomous behaviour was met with unpleasant reactions from the significant elders. Below she talks about the consequences of this lack of ‘obedience’.

*My mother is not saying anything anymore because she’s saying I don’t listen to her so she wants nothing to do with me. I will sort myself out but I don’t care what they say I’m just waiting for the right months to feed him solids. She is angry that I didn’t agree to feed him porridge when she advised me so at three weeks. Now she’s angry she’s saying “the baby will not grow he will trouble you and you will always carry him”. She just says she fed porridge to her babies and that how they grew up and nothing wrong happened, and there’s nothing wrong with it. But I don’t see it that way, time has changed, it not like before and these porridges have so many things in it, so how would I know which one is right (Teen 4, rural_month5).*

### Autonomy and teenage dependency

Generally, mothers consulted and accepted any feeding advice in the household without examining or challenging it because elders were perceived to have more knowledge and more experience with regards to baby care, as stated by teen 10:

*I: Mm…there are many outlets let’s say that talk about ways to feed the baby like magazines, internet and mom-connect [DoH sms support programme]. Have you ever consulted one of those if maybe you want to know something about what to feed your baby…?*

*P: No I consult my mom, she’s got 3 kids, and she knows better (Teen 10, urban_month1)*

Teen mothers mostly depended on their elders for financial support as illustrated in Table 1. Further, they depended on elders for also guidance and experience.

*I: The box has instructions that tells you how to prepare the milk, do you follow those instructions?*

*P: I never read them because my mother tells me what to do, (Teen 8, urban_month5)*

This behavior by teenagers illustrated the extent of dependency they had on their elders, which meant that most of the time teenagers applied certain advice from the elders with no understanding of the purpose. For instance, teen 2 stated:

*My grandmother told me to feed him this type of formula, I don’t know why she said that. (Teen 2, rural_month2)*

Participants also emphasized that in addition to them ‘trusting’ advice from the elders, they also witnessed immediate results in the babies’ behavior, for example, a baby sleeping for longer. This led to mothers being receptive of further suggestions as they viewed them as a way of the family ‘helping out’. However, a few mothers stated that they were not happy with some solids given to the baby but this was never communicated with the elders. This teenage mother narrated this:

*I had a problem with porridge because he was still young and that I shouldn’t feed him but I fed him anyway. What was I supposed to do because my mum said I must feed him? But I didn’t like to and I fed him anyway. He was too young to be fed maize porridge (Teen 8, urban_month6).*

### Managing conflict between clinic advice vs. home advice

When participants were asked about their main source of infant feeding advice, they frequently referred to advice given at home*. “My aunt who can tell me something and I listen” (Teen 3, rural_month3).*

Further, this study revealed that when teenagers were faced with a conflict about whether to accept advice from clinic staff or from family members at home, they usually chose advice from home. The instances mentioned by participants where they chose advice from home included how to prepare formula, introducing solids and use of traditional medicine. Teen 9 below narrates a time when she was faced with such conflicting advice:

*They said at the clinic we must reduce food for her and my mother was like ‘no let us not reduce food because she’s going to be hungry, let us reduce milk’. So, yah making decisions while choosing the nurses, listening to her, yah it was confusing. I did not know if I trust my mum and what my mum says or not. It was very confusing my mom would tell me something else, the nurses would tell me something else. But I actually listened to my mum. I just end up listening to my mum (Teen 9, urban_ month6).*

All teenagers were exposed to infant feeding education during ANC. This meant that when choosing to take advice from home, they were aware that they were not complying with recommended feeding practices. However, even with this knowledge, teenagers were not empowered to apply health workers’ feeding advice. At times teenagers avoided conflict during clinic visits by not revealing the truth about what the baby eats when asked by health workers. One teen reported:

*P: Last week I took him here to the mobile clinic and when they asked me what I am feeding the baby I told them that I feed him breast milk. I didn’t say I also give him porridge, she then said I must not stop and must continue giving him breast milk (Teen 5, rural_month5)*

### Poor feeding advice from key family members

This study revealed that most poor feeding practices adopted by teen mothers were suggested by key role-players in the household. Traditional medicine and water were amongst the fluids most frequently suggested by grandmothers, and these were perceived by elders to help with cleaning the babies’ intestines. Also, most elders perceived a baby crying as an indication of hunger and as a result solids or formula were suggested for better health and better sustenance. Teen 1 explains: *“Mom was like ‘no we won’t sleep today’ cos she kept screaming like my breast milk was not enough for her” (Teen9, urban_month1).*

The kind of solid food suggested was mainly maize meal porridge and this was suggested as early as in the first month of a baby’s life.

*I: Ok, he’s 6 weeks, alright. Now that you are feeding him porridge who gave you that advice?*

*P: My mother said I can feed him porridge because he was not getting full (Teen 7, urban_month2)*

Another teenager from an urban area mentioned:

*He was 2 months when my mum said I must cook maize porridge for him because with solids he would be full for a long time and I fed him. After three months she said I must feed him other solids, I gave him. I would go and buy food for him, then I would feed him. Now that he’s…he’s grown a bit she said I must make mash potatoes, sometimes butternut and cook fruit when it has softened up I shall blend it and feed him (Teen 8, urban_month6).*

### Returning to school

Seven out of ten teenage mothers in the study returned to school within the first month of giving birth. Three of them continued to breastfeed by expressing breastmilk to feed the baby, while most started introducing formula feeds in addition to breastfeeding once they went back to school. Teen 3, who expressed breastmilk for her baby upon returning to school, stated:

*P: I’m breastfeeding him and when I go to school I leave him with expressed milk. I express on his bottle and in a cup, then I put it in a fridge because I am at school for long period. My mom would then take it out when I’m at school and warm it using warm water (Teen 3, rural_month1).*

The same teen was asked about how she knows about the expression methods, she stated:

*P: The lactation advisor [lactation counsellor at the hospital]. He told me that I must express for 6 months. I can put the milk in the fridge or in the freezer for a long period. I followed his instruction. He also said if I want that thing to express [breast pump] I must go and check it at [name of shops], that is where I could find it. (Teen 3, rural_month1)*

When we visited the same mother at 2 months, she was found to be mixed feeding and she stated that being unable to express enough milk to leave behind to feed her baby as the reason for this.

*P: I feeding him both because sometimes breast milk doesn’t come out enough so I thought it better to mixed feed him (Teen 3, rural_month2)*

Some teens, especially those coming from the urban area, mentioned being uncomfortable with breastfeeding in public. This led them to feed their babies formula milk in public places. Teen 9 revealed:

*P: I don’t like to take out my breasts in public and feed the baby, I don’t like it. I can’t, but I do see some other people doing it but no I cannot imagine myself doing it, so I give him formula (Teen 9_urban_month3).*

## Discussion

This study prospectively explored autonomy and infant feeding decision making among teenage mothers from a rural and urban setting in KZN, South Africa. To our knowledge, this is the first study to investigate autonomy and infant feeding decisions for teenage mothers in our context. Our findings revealed that teenage mothers were knowledgeable about the benefits of EBF and most teenagers planned to EBF for 6 months based on counselling received during ANC and post-delivery. Although all the mothers who planned to EBF did initiate breastfeeding, they were not able to sustain it for the full 6 months. This was because, once participants returned home after giving birth, responsibility for caring for the infants’ needs was handed over to older members of the family. These elders were not supportive of EBF and frequently suggested starting complementary feeding as early as within the first month. Furthermore, once teenagers returned to school, they were unable to maintain breastfeeding and when given conflicting feeding advice from the clinic and home, they usually chose home advice.

All teenagers were unmarried and lived with immediate or extended family members, upon whom most were dependent for support and childcare guidance as most teenage participants were having their first child. The data revealed that, among teenagers who followed feeding advice from elders, this advice was perceived as helpful and a positive support. Similar findings have been reported in the study conducted by Devito and colleagues [11], where it was found that teenage mothers usually identify their own parents as their main source of support. A key finding from our study, was that most teenage mothers from both urban and rural settings were not empowered to reject the feeding choices made in the household, although they knew these to be against the recommended feeding practices. This demonstrates the lack of autonomy that participants had within the household, as they were unable to voice their concerns. Autonomy can be understood as one’s freedom to regulate their own behavior as part of the process of developing independence and self-guided action [26].

In an African household, the concept of autonomy and decision making for teenagers should be viewed through the lenses of ‘epistemological authoritarianism’, a concept found in the writings of Kaphagawani [27]. According to Kaphagawani, in African households, the adolescent stage is linked with incompleteness. This view reinforces the notion that children need understanding and adult help. Further, adolescence is associated with lack of autonomous thinking, where young individuals are perceived as less competent in dealing with serious life issues, such as child care. As a result, the “child’s voice in an adult-child relationship becomes silenced and invisible” [27].

The diminished autonomy was noted consistently from both urban and rural settings, with the exception of one teenager who was slightly older than the rest of the participants and was having her second child. Lack of autonomy and decision making power for young mothers has been identified by other authors in other settings [8, 28]. It is vital for health care professionals to understand the context in which infant feeding decision occurs when working with teenagers. For mothers who were able to be autonomous in their decision making, such as Teen 4, withdrawal of support from parents and severe pressure by family to add other food were noted. Programmes aimed at empowering this vulnerable population to apply advice from the health workers on optimal feeding practices are needed, and these should include strong participation of key family members, like the child’s grandmother. Leerlooijer et al. give an example of such a programme which is implemented in rural Uganda, where unmarried teenage mothers’ psychological and social well-being is enhanced by increasing their decision making power and creating a supportive environment [29].

The most common feeding practice reported by participants that was encouraged by family members, was the early introduction to the infant’s diet of other food or fluids, a practice which may put infants at risk of diarrhoeal disease and malnutrition. This is similar to the findings of Devito and colleagues, who reported that adolescent mothers participated in unhealthy infant feeding practices because of inaccurate or inadequate advice from family members [11]. Poor nutrition has been cited to contribute to almost half of child deaths globally in 2016 [30]. Studies have shown that teenage mothers and their children are more prone to having disadvantages such as malnutrition in the long term due to lack of preparation for motherhood and lack of maturity [6, 31].

Given that the advice which influenced teenage mothers’ feeding choices was poor and did not comply with the current SA Department of Health infant feeding policy [32], it is important for infant feeding interventions to include close family members as the integral part of infant feeding counselling from ANC throughout infancy. This approach could result in the support given to teen mothers in the household being in line current infant feeding recommendations [32]. Currently the ANC is mainly aimed at mothers, thus focusing the education on the teenagers only. A similar recommendation was made by Fjeld and his colleagues, who also suggested the importance of targeting teens together with their family members when promoting EBF [28]. In addition, the environment, which extends beyond the household where breastfeeding occurs, needs to be improved to provide a context that encourages breastfeeding and this requires a multi- faceted intervention.

Factors such as returning to school and fear of breastfeeding in public led to mothers introducing other foods and liquids before their baby reached 6 months. Strategies such as expressing milk into a cup for mothers to take with when travelling with the baby, need to be particularly emphasized by health care workers for this population, as they were more likely than older age groups to be affected be the stigma related to breastfeeding in public and opted for formula feeding when going out with the baby. Broader societal interventions should also aim at improving the image of breastfeeding to overcome this challenge.

It is, however, important to note that although expressing is advised as the best short-term solution for maintaining exclusive breastfeeding when the mother is away from the baby, it may be a challenge for school going mothers, as they need to express frequently in order to maintain milk supply [33]. This is due to the fact that school facilities in South Africa are currently not equipped to support breastfeeding school children. Therefore, appropriate interventions aimed at the facility-based level need to be designed to support lactating school children as suggested by the SA Minister of Health [34]. These can include providing a room with a fridge to store breastmilk at school where babies are cared for and where mothers can go during breaks to breastfeed.

Limitations of the study include that, as a result of the longitudinal design, all mothers were recruited at a similar time point and we were therefore unable to sample to saturation at the outset. However, analysis continued until no new themes emerged among our participants suggesting that saturation was achieved. In addition, our results appear to be context specific, and the qualitative methodology employed means that our results are not generalizable. However, the consistency of the findings among our participants suggests that these have a broad application to teenagers in our setting, and other studies support lack of autonomy among teenagers as a factor to be considered when supporting breastfeeding teenagers in other contexts.

## Conclusion

Suboptimal infant feeding practices have been reported for younger mothers, including this study which highlighted the critical and large role of key family members, by whom teenagers’ infant feeding choices are most influenced. Our findings suggest that to improve nutritional status of children in our setting, the cultural values which guide the infant feeding decision making process in African households should be considered. Thus, a comprehensive, socio-cultural approach which, moves beyond focusing on the mother alone when promoting optimal feeding practices is likely to succeed in our complex context.

## Abbreviations

ANC: Antenatal care
DHSI: District Health Information System
DoH: Department of Health
EBF: Exclusive Breastfeeding
KZN: KwaZulu-Natal
SA: South Africa
WHO: World Health Organization
